# Liver transplantation for giant hemangioma of the liver: A case report and review of the literature

**DOI:** 10.3389/fmed.2022.985181

**Published:** 2022-09-15

**Authors:** Yun Zhao, Xiu-Ping Li, Yuan-Yuan Hu, Ji-Chang Jiang, Li-Jin Zhao

**Affiliations:** Department of General Surgery, Digestive Disease Hospital, Affiliated Hospital of Zunyi Medical University, Zunyi, China

**Keywords:** liver transplantation, cavernous hemangioma, giant hemangioma, hepatic hemangioma, case report

## Abstract

Large benign hepatic masses as a rare indication for liver transplantation have been reported less frequently. These liver transplantations are complex, with high intraoperative bleeding, high perioperative complication rates, and high mortality rates due to difficulties in visualization, especially when they have undergone various percutaneous operations or open surgery, resulting in severe perihepatic adhesions. Here is a case report of a patient admitted to our hospital who underwent liver transplantation after suffering from a giant hemangioma in liver transplantation for 10 years and who had received multiple interventional treatments ineffective in the past.

## Introduction

Hepatic hemangioma (HH) is the most common benign tumor of the liver. It is usually considered to be a vascular malformation, possessing a vascular nature with a flattened endothelial cell lining, probably related to excessive development or differentiation during embryonic development, and manifesting as well-defined vascular-rich lesions. Most HH originates from the right lobe of the liver, and its pathophysiology has not been clearly elucidated. HH can be divided into subtypes such as cavernous hemangioma, capillary hemangioma, epithelioid hemangioendothelioma, and sclerosing hemangioma according to the amount of fibrous components contained in the tumor, among which hepatic cavernous hemangioma is the most common. Herein, we report a case of a 9-kg giant HH that underwent liver transplantation and evaluate the available literature.

## Case report

**FIGURE 1:**
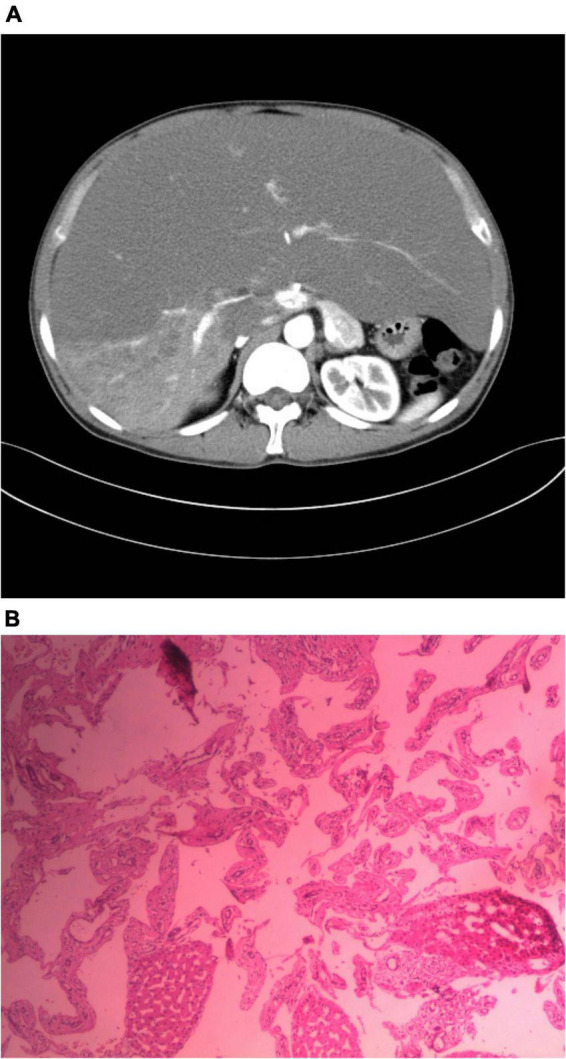
**(A)** CT scan of the lesion. **(B)** Pathological images.

**FIGURE 2:**
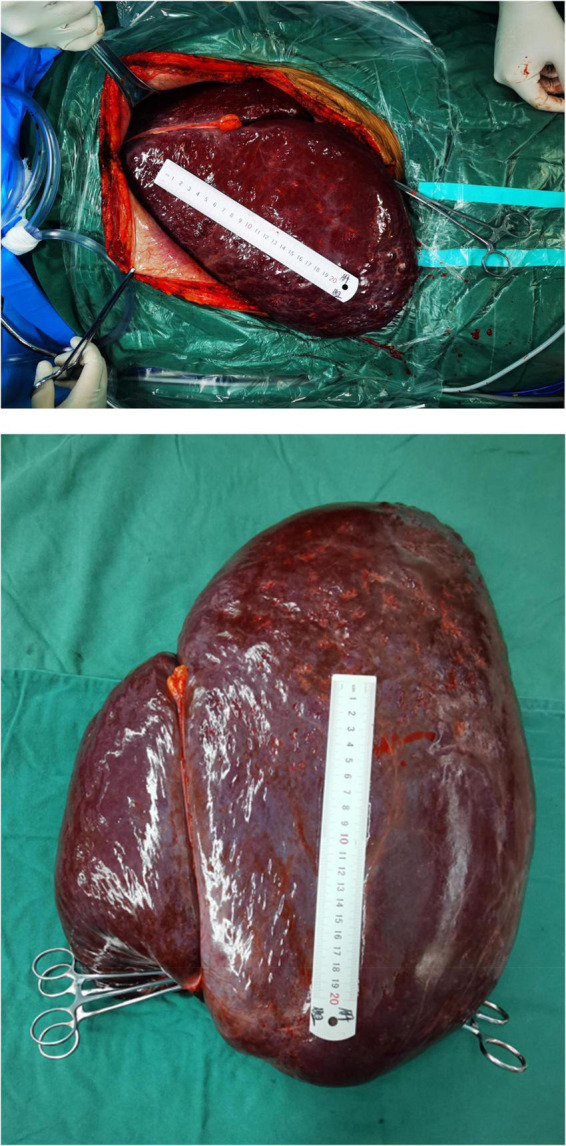
Intraoperative images.

A male patient, 36 years old, was admitted to the hospital with “a giant hemangioma of the liver found 10 years ago.” The patient was diagnosed as having a giant hemangioma of the liver after he was examined at our hospital 10 years ago for abdominal distention, with no apparent cause, which worsened after eating, and occasional abdominal pain, diarrhea, nausea, and vomiting. At that time, the patient refused surgical resection treatment for personal reasons, and, instead, underwent several interventions to relieve the disease, but the results were not good, and then the patient was admitted. Physical examination: There was no yellow staining of the skin and sclera, no liver palm or spider nevus, and no enlargement of superficial lymph nodes throughout the body. The liver was palpable under the ribs, with the inferior hepatic margin located 15 cm below the intersection of the right midclavicular line and the right rib margin, and 10 cm below the glabella, soft, non-tender, with rounded edges, a smooth surface and no nodules, and the spleen was not palpable under the ribs. There was no percussion pain in the liver area, mobile turbid sound (-), Murphy’s sign (-), normal bowel sounds, and no edema in both lower limbs. Pre-transplantation ancillary examinations: routine blood, coagulation function, liver and kidney function, electrolytes, blood sedimentation, tumor markers, calcitoninogen, and other test results did not show significant abnormalities. Enhanced CT of the upper abdomen: the liver was significantly enlarged, and the lower edge reached the right iliac fossa, suggesting a giant hemangioma of the liver (Figure 1A). The clinical diagnosis was: giant hemangioma of the liver. The patient’s MELD score and the Child-Pugh score were 13 and 7, respectively, due to the unsatisfactory results of multiple interventions for the giant hemangioma and the inability of surgical resection, and the patient was finally placed on the liver transplantation waiting list after discussion by our transplantation team. After perfecting the preoperative preparation, we performed a classical *in situ* whole liver transplantation with a good quality donor liver, and the hypothermic ischemia time, warm ischemia time, and anhepatic phase were 165, 2, and 63 min, respectively, for a total operative time of 385 min. The entire liver and the hemangioma were completely removed (Figure 2), and a new liver was implanted, and the transplantation was successfully completed. The postoperative pathology revealed that the tumor consisted mainly of a large number of abnormally dilated blood sinuses, suggesting a hepatic cavernous hemangioma (Figure 1B). Postoperatively, the tracheal intubation was successfully removed, and the patient was conscious and safely returned to the liver transplantation ward. The immunosuppressive regimen was morte-macrolimus and tacrolimus. Normal diet was resumed on postoperative Day 3, and the patient was discharged on postoperative Day 13. At regular follow-up for 6 months, the graft survived well, liver and renal function and electrolytes were in the normal range, and the patient had a good quality of life.

## Discussion

With the gradual increase in the understanding of HH and the rapid development of imaging techniques, the detection rate of HH has been increasing, with a reported incidence of 0.4–20% and a male-to-female ratio of approximately 1:5 (1), and further studies have shown (2, 3) that hemangiomas in menopausal women on estrogen replacement therapy and pregnant women have a rapid growth rate and a high risk of secondary rupture, and such results suggest that the cause of such gender differences may be related to the sex hormones in women.

HH is usually asymptomatic and is detected only during physical examination, dissection, or autopsy. Approximately, 61% of patients have small, solitary lesions that grow slowly and have a long course, and patients may have no significant abnormalities in liver function (4). Generally, HH with a diameter <4 cm is asymptomatic, but, if the tumor diameter is >4 cm, a series of symptoms and signs may appear due to the space occupation of the abdominal cavity by the larger tumor and the compression of the surrounding tissues, with pain or fullness in the right upper abdomen being the most common complaint, and, occasionally, loss of appetite, nausea, and vomiting due to compression of the gastrointestinal tract by the left giant HH. This may lead to jaundice or compression of the inferior vena cava or hepatic vein, leading to Buga syndrome. Very rarely, giant HH may lead to severe coagulation abnormalities, such as kamagra syndrome, which manifests as hemolytic anemia, thrombocytopenia, prolonged prothrombin time, and hypofibrinogenemia (5). In addition, there are less common symptoms, including fever, dyspnea, high-output heart failure, and biliary bleeding (6).

The diagnosis of HH currently relies on imaging, and the unique features of HH imaging are the presence of peripheral nodal enhancement and progressive centripetal filling, with ultrasound (US), computed tomography (CT), and magnetic resonance imaging (MRI) being the most commonly used examinations. Among them, US is the preferred method of examination and presents as a well-defined, homogeneous hyperechoic mass that can be deformed under pressure, and larger hemangiomas show mixed echogenicity, with internal echoes still predominantly hyperechoic. Although US is more sensitive, its specificity is only 60.3% because it only highlights blood flow and intra-arterial portal shunts in HH, and some malignant liver lesions (hepatocellular carcinoma intrahepatic metastases, etc.) may also produce similar acoustic patterns (7, 8). HH is routinely scanned by CT with a plain scan and enhancement (the commonly used contrast agent is iodine), and the plain scan presents a well-defined, uniformly dense when contrast is used, a centripetal, uniformly filled peripheral nodular enhancement with “fast in and fast out” is characteristic, but small lesions (<3 cm), cystic areas, fibrosis or thrombosis may show an atypical pattern (6). Compared with US and CT, MRI has the highest sensitivity and specificity in the diagnosis of HH, with a low signal on T1-weighted imaging and a high signal on T2-weighted imaging, with uniform intensity and clear borders, and increasing signal intensity with prolonged echo time, and a higher signal on heavy T2-weighted imaging, which is called the “light bulb sign”; intra-tumor thrombus, the enhancement pattern of the dynamic scan in MRI, is similar to that of CT, which is “fast-in, slow-out.” In addition, the weak signal on T1-weighted imaging and the high-intensity signal on T2-weighted imaging are important features for differentiation from hepatocellular carcinoma (9), while angiography is the best choice for atypical HH that is difficult to diagnose with other imaging examinations, which is manifested as the “snow tree sign” or the “cotton wool sign” (6). In addition, fine-needle aspiration biopsy is not recommended due to the high risk and low diagnostic yield associated with bleeding.

HH is asymptomatic in most cases and has no malignant tendency, so, in principle, no treatment is needed, and follow-up observation is the main focus. Treatment should be limited to patients with complications, such as symptoms, persistent enlargement of the mass, compression of adjacent organs (Gastric outlet obstruction, Budd-Chiari syndrome), hemangioma rupture, and bleeding or Kasabach-Merritt syndrome (KMS), which is an internationally accepted concept, and its treatment includes radiofrequency ablation, hepatic artery interventional embolization, surgical resection, and liver transplantation (10). Although the effectiveness of interventional treatment for this tumor has been well documented, unfortunately, this modality is only temporarily effective in reducing the size of the hemangioma and reoccurs with revascularization, followed by the reappearance of a series of symptoms, such as coagulation disorders. Although liver transplantation for benign tumors is not used as a first-line treatment, in this case, the tumor was huge and seriously affected the patient’s life, and the results of multiple interventional procedures performed in the past were poor, and surgical resection may cause uncontrollable hemorrhage and serious complications, such as small liver syndrome and liver failure, after resection of the giant tumor. It was also concluded from a review of the relevant literature that surgical pointers for liver transplantation for HH include respiratory distress due to giant HH, persistent abdominal pain and discomfort, diffuse masses or masses growing rapidly with risk of rupture, KMS, diffuse intravascular coagulation, and failure of previous interventions, excluding liver transplantation. To date, to the authors’ knowledge, there are currently 19 case reports of liver transplantation for giant hepatic hemangiomas in 18 studies, of which the youngest patient was only 28 days old, and the oldest patient was 56 years old, and the majority of liver transplants were due to KMS, with other causes including respiratory distress due to a large mass (*n* = 4), persistent abdominal distension, abdominal pain (*n* = 3), thrombosis due to compression of the inferior vena cava (*n* = 2), and bleeding from ruptured mass (*n* = 1), as detailed in Table 1.

**TABLE 1:** Current literature on liver transplantation for hepatic hemangioma.

References	Age (years)/ sex	Clinical preoperative symptoms	Graft type	LH size (cm)	Re-LT	Follow-up	Complications	Condition
Klompmaker et al. (11)	27/M	KMS	CD/whole	N/A	No	3 years	Uneventful	Alive
Mora et al. (12)	42/F	KMS	CD/whole	N/A	No	16 days	N/A	Alive
Tepetes et al. (13)	4 weeks/M	KMS	CD/whole	N/A	No	8 days	Abnormal graft function; intracerebral hemorrhage	Died
Chui et al. (14)	1.33/F; 2.43/F	1. Epigastric pain, bloating, exertional dyspnea; 2Progressive hepatomegaly with abdominal discomfort	CD/whole	1. N/A; 2.40 × 35 × 15	1. Yes; 2. No	1.18 mouths; 2.14 mouths	1. Intraoperative massive bleeding, acute renal failure, re-transplantation; 2. uneventful	Both Alive
Longeville et al. (10)	47/M	KMS	CD/whole	35	N/A	1 years	Internal bleeding after transplantation	Alive
Russo et al. (15)	43/F	N/A	CD/whole	21	N/A	N/A	N/A	Alive
Keegan et al. (16)	34/M	Respiratory distress, persistent abdominal distension, abdominal pain	CD/whole	63 × 45 × 51	No	1 years	Postoperative abdominal bleeding, open abdomen to stop bleeding	Alive
Kumashiro et al. (17)	48/F	KMS, acute liver failure	LD/posterior lobe	N/A	N/A	N/A	Intraoperative massive bleeding	Alive
Ferraz et al. (18)	25/F	KMS, breathing distress	CD/whole	46 × 40 × 15	N/A	30 mouths	Acute rejection	Alive
Meguro et al. (19)	45/F	KMS	LD/left lobe	15	N/A	10 mouths	Intraoperative massive bleeding, acute rejection (post-operative day 8), small size graft syndrome, sepsis (post-operative day 31)	Alive
Aseni et al. (20)	46/M	Recurrent pulmonary embolism due to inferior vena cava compression	CD/whole	N/A	No	25 mouths	N/A	Alive
Vagefi et al. (21)	32/F	Rupture, KMS	CD/whole	18 × 23	No	N/A	Uneventful	Alive
Unal et al. (22)	56/F	KMS	CD/whole	N/A	No	6 mouths	Uneventful	Alive
Yildiz et al. (23)	44/F	KMS, Breathing distress	CD/whole	22 × 18 × 23	No	1 mouths	Uneventful	Alive
Zhong et al. (24)	27/F	Upper abdominal discomfort	LD/right lobe without middle hepatic vein	50 × 40 × 25	No	17 mouths	Acute rejection	Alive
Lange et al. (25)	46/F	Portal vein thrombosis, ascites, deep vein thrombosis and pulmonary thromboembolism	CD/whole	21.7 × 23.7 × 25.5	No	7 weeks	Uneventful	Alive
Lee et al. (26)	51/F	Compression symptoms caused by a rapidly growing liver	LD/modified right lobe	N/A	No	16 weeks	Uneventful	Alive
Eghlimi et al. (5)	38/M	Progressive abdominal distention, difficulty swallowing	CD/whole	32.4 × 26 × 3.1	No	8 mouths	Cytomegalovirus infection	Alive

F, Female; M, Male; KMS, Kasabach-Merritt Syndrome; CD, Cadaveric; LD, Living donor; N/A, Not available.

Although surgical resection is still the most effective treatment for hepatic hemangioma, hepatectomy requires consideration of the residual function of the liver, the condition of the liver, the size and the number of tumors, and the location of the tumor. Considering that the tumor occupies almost the whole liver and the liver residue is insufficient after resection, which may lead to small liver syndrome, plus the fact that the patient’s interventions have been ineffective for many times since the patient’s illness, and the symptoms have been worsening, with the possibility of rupture and compression symptoms, which are indicative of liver transplantation, liver resection was not considered, and the patient was placed on the liver transplantation waiting list.

It is noteworthy that, in this case, due to the large size of the diseased liver, it was difficult to reveal it. In order to fully expose the operative field, the surgical incision should be large enough, so we chose a “herringbone” incision in the upper abdomen, which was about a 40-cm long, and then entered the abdominal cavity layer by layer. The lower edge of the liver reached the pelvic cavity. The huge liver was clearly adherent to the surrounding intestinal tract, and a clearly edematous gastrointestinal tract could also be seen, which posed considerable difficulties in continuing the operation. In this case, the first hepatic hilar and inferior hepatic vena cava were preemptively blocked to avoid hemorrhage when the liver was freed. By blocking the blood flow to the liver, the size of the HH was gradually reduced, thus allowing full exposure of the operative field. Meanwhile, in addition to the important role of surgical technique and experience in limiting blood loss, intraoperative venous bleeding can be reduced by lowering central venous pressure (CVP) and portal vein pressure, thereby reducing collateral vessel filling, which, in turn, helps to reduce intraoperative venous bleeding. Methods to reduce CVP include fluid volume restriction, venous dissection, use of diuretics, such as mannitol, low tidal volume ventilation, and avoidance of high positive end-expiratory pressure ventilation. The potential benefits of lowering CVP should outweigh the risk of inadequate organ perfusion and renal injury to the patient. The need to meet tissue oxygen requirements while reducing CVP can be assessed by measuring mixed venous oxygen saturation, arterial blood lactate, and other parameters. Intraoperative bleeding in liver transplantation is closely related to the severity of the disease, coagulation, and surgical proficiency. The first thing to note is the patient’s coagulation status, while changes in prothrombin time and activated partial thromboplastin time have limited significance in determining coagulation function intraoperatively. Intraoperative thromboelastography (TEG), which dynamically reflects changes in blood coagulation status, can be used to monitor the patient’s coagulation profile to assess coagulation function, guide the use of medications and evaluate their effects, avoid massive blood transfusions due to disorders in coagulation mechanisms, and reasonably transfuse blood products. These include suspended red blood cells, fresh frozen plasma and platelets. In addition, intraoperative blood retrieval transfusions can be performed by collecting the recipient’s own blood or retrieving blood lost in the intraoperative field to meet his or her own perioperative blood supply. Intraoperative recovered autologous transfusion uses a negative pressure suction device to collect blood lost during surgery, and then returns it to the patient after filtering, separation, washing, purification, and concentration. Autologous blood transfusion can not only save limited blood resources and reduce the economic burden of patients but also avoid a series of adverse reactions and potential infectious diseases caused by massive transfusion of allogeneic blood during liver transplantation.

After complete resection of the diseased liver, we were faced with the challenge that the donor liver was relatively small compared to the diseased liver, and the length of vessels and bile ducts required for implantation of the new liver had to be adequate for transplantation. The duodenal ligament can be dissected as close to the liver as possible in order to preserve sufficient length of vessels and bile ducts, and, in view of the large difference in size between the diseased liver and the donor liver, the liver may be displaced after implantation. To prevent this, the perihepatic ligament was further sutured and secured in the anterior abdomen during surgery, and intraoperative ultrasound was used to determine the patency of the vessels and bile ducts. In addition, we believe that resection of a large diseased liver is often difficult, and there are a number of uncertainties that can lead to prolonged operative times. Firstly, the prolonged duration of the operation to remove the diseased liver leads to prolonged hypothermic ischemia time of the donor liver. Secondly, uncontrollable hemorrhage during resection of the diseased liver requires rapid removal of the diseased liver, which can lead to a prolonged anhepatic phase if the donor liver has not yet been repaired. Thirdly, dissection of the diseased liver is difficult, and the operation takes longer. Therefore, when facing this type of surgery, not only do we need to be familiar with the anatomical location and cooperate carefully intraoperatively, but, also, the donor liver acquisition, diseased liver resection, and recipient surgery teams should communicate in a timely manner and actively improve the preoperative preparation so that the donor and recipient surgery can be more closely connected to facilitate successful surgery.

## Conclusion

Although the best treatment for giant HH is not yet uniform, liver transplantation has been shown to be a safe and effective treatment in cases where conservative treatment is ineffective and surgical resection is not possible.

## Data availability statement

The original contributions presented in this study are included in the article/supplementary material, further inquiries can be directed to the corresponding author.

## Ethics statement

Written informed consent was obtained from the individual(s) for the publication of any potentially identifiable images or data included in this article.

## Author contributions

YZ was responsible for formulating writing ideas and writing the manuscript. X-PL, Y-YH, and J-CJ were responsible for collecting information. L-JZ was responsible for revising the manuscript and guiding the writing of the manuscript. All authors contributed to the article and approved the submitted version.

## References

[1] Prodromidou A Machairas N Garoufalia Z Kostakis ID Tsaparas P Paspala A Liver transplantation for giant hepatic hemangioma: a systematic review. *Transplant Proc.* (2019) 51:440–2. 10.1016/j.transproceed.2019.01.018 30879561

[2] Donati M Stavrou GA Donati A Oldhafer KJ. The risk of spontaneous rupture of liver hemangiomas: a critical review of the literature. *J Hepatobiliary Pancreat Sci.* (2011) 18:797–805. 10.1007/s00534-011-0420-7 21796406

[3] Gupta S Agarwal V Acharya AN. Spontaneous rupture of a giant hepatic hemangioma-report of a case. *Indian J Surg.* (2012) 74:434–6. 10.1007/s12262-011-0309-3 24082605 PMC3477415

[4] Dong J Zhang M Chen JQ Ma F Wang HH Lv Y. Tumor size is not a criterion for resection during the management of giant hemangioma of the liver. *Eur J Gastroenterol Hepatol.* (2015) 27:686–91. 25923944 10.1097/MEG.0000000000000344

[5] Eghlimi H Arasteh P Azade N. Orthotopic liver transplantation for management of a giant liver hemangioma: a case report and review of literature. *BMC Surg.* (2020) 20:142. 10.1186/s12893-020-00801-PMC7324977 32600292

[6] Leon M Chavez L Surani S. Hepatic hemangioma: what internists need to know. *World J Gastroenterol.* (2020) 26:11–20. 10.3748/wjg.v26.i1.11 31933511 PMC6952297

[7] Lim KJ Kim KW Jeong WK Kim SY Jang YJ Yang S Colour doppler sonography of hepatic haemangiomas with arterioportal shunts. *Br J Radiol.* (2012) 85:142–6. 21385916 10.1259/bjr/96605786 PMC3473947

[8] Toro A Mahfouz AE Ardiri A Malaguarnera M Malaguarnera G Loria F What is changing in indications and treatment of hepatic hemangiomas. A review. *Ann Hepatol.* (2014) 13:327–39. 24927603

[9] Liu X Yang Z Tan H Liu L Xu L Sun Y Characteristics and operative treatment of extremely giant liver hemangioma >20 cm. *Surgery.* (2017) 161:1514–24. 10.1016/j.surg.2016.12.015 28126252

[10] Longeville JH De La Hall P Dolan P Holt AW Lillie PE Williams JA Treatment of a giant haemangioma of the liver with kasabach-merritt syndrome by orthotopic liver transplant a case report. *HPB Surg.* (1997) 10:159–62. 9174860 10.1155/1997/10136 PMC2423854

[11] Klompmaker IJ Sloof MJ Van Der Meer J de Jong GM de Bruijn KM Bams JL. Orthotopic liver transplantation in a patient with a giant cavernous hemangioma of the liver and kasabach-merritt syndrome. *Transplantation.* (1989) 48:149–51. 10.1097/00007890-198907000-00035 2501918

[12] Mora A CortéS C Roigé J Noguer M Camps MA Margarit C. [Orthotopic liver transplant for giant cavernous hemangioma and kasabach-merritt syndrome]. *Rev Esp Anestesiol Reanim.* (1995) 42:71–4. 7899656

[13] Tepetes K Selby R Webb M Madariaga JR Iwatsuki S Starzl TE. Orthotopic liver transplantation for benign hepatic neoplasms. *Arch Surg.* (1995) 130:153–6. 7848084 10.1001/archsurg.1995.01430020043005

[14] Chui AK Vass J Mccaughan GW Sheil AG. Giant cavernous haemangioma: a rare indication for liver transplantation. *Aust N Z J Surg.* (1996) 66:122–4. 10.1111/j.1445-2197.1996.tb01132.x 8602810

[15] Russo MW Johnson MW Fair JH Brown RS Jr. Orthotopic liver transplantation for giant hepatic hemangioma. *Am J Gastroenterol.* (1997) 92:1 940–1. 9382077

[16] Keegan MT Kamath GS Vasdev GM Findlay JY Gores GJ Steers JL Liver transplantation for massive hepatic haemangiomatosis causing restrictive lung disease. *Br J Anaesth.* (2001) 86:431–4. 10.1093/bja/86.3.431 11573537

[17] Kumashiro Y Kasahara M Nomoto K Kawai M Sasaki K Kiuchi T Living donor liver transplantation for giant hepatic hemangioma with kasabach-merritt syndrome with a posterior segment graft. *Liver Transpl.* (2002) 8:721–4. 10.1053/jlts.2002.33689 12149767

[18] Ferraz AA Sette MJ Maia M de Almeida Lope EP Godoy MMG Petribu ATDS Liver transplant for the treatment of giant hepatic hemangioma. *Liver Transpl.* (2004) 10:1436–7. 15497149 10.1002/lt.20250

[19] Meguro M Soejima Y Taketomi A Ikegami T Yamashita Y Harada N Living donor liver transplantation in a patient with giant hepatic hemangioma complicated by kasabach-merritt syndrome: report of a case. *Surg Today.* (2008) 38:463–8. 10.1007/s00595-007-3623-4 18560973

[20] Aseni P Lauterio A Slim AO Giacomoni A Lamperti L De Carlis L. Life-saving super-urgent liver transplantation with replacement of retrohepatic vena cava by dacron graft. *HPB Surg.* (2010) 2010:828326. 10.1155/2010/828326 20811479 PMC2926580

[21] Vagefi PA Klein I Gelb B Hameed B Moff SL Simko JP Emergent orthotopic liver transplantation for hemorrhage from a giant cavernous hepatic hemangioma: case report and review. *J Gastrointest Surg.* (2011) 15:209–14. 20549381 10.1007/s11605-010-1248-1 PMC3023038

[22] Unal E Francis F Aquino A Xu R Morgan G Teperman L. Liver transplant for mixed capillary-cavernous hemangioma masquerading as hepatocellular carcinoma in a patient with hepatocellular carcinoma. *Exp Clin Transplant.* (2011) 9:344–8. 21967263

[23] Yildiz S Kantarci M Kizrak Y. Cadaveric liver transplantation for a giant mass. *Gastroenterology.* (2014) 146:e10–1. 10.1053/j.gastro.2013.08.001 24269562

[24] Zhong L Men TY Yang GD Gu Y Chen G Xing TH Case report: living donor liver transplantation for giant hepatic hemangioma using a right lobe graft without the middle hepatic vein. *World J Surg Oncol.* (2014) 12:83. 10.1186/1477-7819-12-83 24708716 PMC4016776

[25] Lange UG Bucher JN Schoenberg MB Benzing C Schmelzle M Gradistanac T Orthotopic liver transplantation for giant liver haemangioma: a case report. *World J Transplant.* (2015) 5:354–9. 26722664 10.5500/wjt.v5.i4.354 PMC4689947

[26] Lee JH Yoon CJ Kim YH Han HS Cho JY Kim H Living-donor liver transplantation for giant hepatic hemangioma with diffuse hemangiomatosis in an adult: a case report. *Clin Mol Hepatol.* (2018) 24:163–8. 10.3350/cmh.2017.0002 28719965 PMC6038937

